# Higher Network Activity Induced by Tactile Compared to Electrical Stimulation of Leech Mechanoreceptors

**DOI:** 10.3389/fphys.2018.00173

**Published:** 2018-03-07

**Authors:** Elham Fathiazar, Gerrit Hilgen, Jutta Kretzberg

**Affiliations:** ^1^Computational Neuroscience, Department of Neuroscience, FK VI, University of Oldenburg, Oldenburg, Germany; ^2^Faculty of Medical Sciences, Institute of Neuroscience, Newcastle University, Newcastle upon Tyne, United Kingdom; ^3^Cluster of Excellence Hearing4all, University of Oldenburg, Oldenburg, Germany

**Keywords:** invertebrate, somatosensory system, touch, pressure, skin stimulation, local bend, interneuron, voltage-sensitive dye imaging

## Abstract

The tiny ensemble of neurons in the leech ganglion can discriminate the locations of touch stimuli on the skin as precisely as a human fingertip. The leech uses this ability to locally bend the body-wall away from the stimulus. It is assumed that a three-layered feedforward network of pressure mechanoreceptors, interneurons, and motor neurons controls this behavior. Most previous studies identified and characterized the local bend network based on electrical stimulation of a single pressure mechanoreceptor, which was sufficient to trigger the local bend response. Recent studies showed, however, that up to six mechanoreceptors of three types innervating the stimulated patch of skin carry information about both touch intensity and location simultaneously. Therefore, we hypothesized that interneurons involved in the local bend network might require the temporally concerted inputs from the population of mechanoreceptors representing tactile stimuli, to decode the tactile information and to provide appropriate synaptic inputs to the motor neurons. We examined the influence of current injection into a single mechanoreceptor on activity of postsynaptic interneurons in the network and compared it to responses of interneurons to skin stimulation with different pressure intensities. We used voltage-sensitive dye imaging to monitor the graded membrane potential changes of all visible cells on the ventral side of the ganglion. Our results showed that stimulation of a single mechanoreceptor activates several local bend interneurons, consistent with previous intracellular studies. Tactile skin stimulation, however, evoked a more pronounced, longer-lasting, stimulus intensity-dependent network dynamics involving more interneurons. We concluded that the underlying local bend network enables a non-linear processing of tactile information provided by population of mechanoreceptors. This task requires a more complex network structure than previously assumed, probably containing polysynaptic interneuron connections and feedback loops. This small, experimentally well-accessible neuronal system highlights the general importance of selecting adequate sensory stimulation to investigate the network dynamics in the context of natural behavior.

## Introduction

Understanding the mechanisms of how sensory information is conveyed onto motor neurons to elicit behavioral reactions is a major goal in neuroscience. Generally, one or several layers of interneurons connect the sensory receptors to motor neurons. Depending on the behavioral context, these interneurons provide synaptic inputs to motor neurons, generating different motor patterns in the same, or overlapping, sets of muscles (Pearson, 1993; Büschges et al., 2011).

The leech nervous system is a useful model to understand the role of interneurons in tactile processing (Wagenaar, 2015). A small sensory-motor network in the leech elicits surprisingly precise behavioral patterns. Leeches produce different motor patterns when locally bending away from stimulus locations that are only separated by 1 mm on their body-wall (Baca, 2005; Kristan et al., 2005).

Leeches have a rigorously segmented nervous system with one ganglion per segment. Each ganglion is an ensemble of around 400 mostly paired neurons, which can be classified into four different functional categories: mechanoreceptors, motor neurons, interneurons and neurosecretory neurons (Wagenaar, 2015). The group of mechanoreceptors contains 14 cells of 3 types, classically assumed to respond to a distinct range of intensities: 6 Touch (T) cells are activated by light touch, 4 pressure (P) cells by a moderate pressure and 4 noxious (N) cells by strong, painful stimuli. Each patch of the skin is innervated by up to 6 of these mechanoreceptors (2 T, 2 P, and 2 N cells). Mechanosensory cells respond to mechanical stimulation of the skin with specific spiking patterns, encoding the location, intensity, and duration of the tactile stimuli (Baca, 2005; Thomson and Kristan, 2006; Kretzberg et al., 2016; Pirschel and Kretzberg, 2016). Interneurons are the largest group of neurons in the ganglion (Nicholls and Baylor, 1968). Most connections from mechanoreceptors to motor neurons are polysynaptic through interneurons (Kristan et al., 2005; Burrell, 2017).

The whole path from sensory input to motor output of the local bend reflex is located in a single ganglion. A three-layered mostly feed-forward network was suggested to control this behavior. Four mechanoreceptors of one type (P cells) as the first layer were concluded to provide the input to the network (Kristan, 1982). A simple interneuronal layer consisting of 25 interneurons receiving synaptic input from all of these 4 P cells (Lockery and Kristan, 1990) was assumed to distribute the sensory input to the appropriate 20 motor neurons in the output layer (Lewis and Kristan, 1998; Kristan et al., 2005), which control the muscle movements.

Intracellular current stimulation of a single P cell elicits fictive muscle movement patterns similar to (but smaller than) local bend responses triggered by touching the skin, while the stimulation of a single T or N cell often elicits only negligible muscle contractions (Kristan, 1982; Zoccolan and Torre, 2002). Therefore, P cells were suggested as the main encoder of tactile stimulus properties and the only relevant input to the local bend network. Until recently, only a single P cell was electrically stimulated to elicit the local bend responses in most of the experiments to study the underlying circuitry (Baljon and Wagenaar, 2015; Frady et al., 2016; Tomina and Wagenaar, 2017).

Recent studies suggested revision of the local bending circuitry (Kretzberg et al., 2016; Pirschel and Kretzberg, 2016; Pirschel et al., 2018). These studies showed that despite the classic association of P and T mechanoreceptor types to distinct stimulus pressure intensities, these cell types respond to stimulus intensities of all ranges. Moreover, all P and T cells carry information about both stimulus intensity and location simultaneously by multiplexing spike counts and spike timing. These findings suggest an operative role of T cells in encoding tactile information (Pirschel and Kretzberg, 2016) and therefore this cell type needs to be considered to provide relevant input to the tactile processing network of the leech. The complex encoding of tactile properties by mechanoreceptors gives rise to the questions of how the encoded tactile information could be decoded by a simple interneuronal layer or whether interneuronal wiring and processing might be more complex than assumed before.

In this study, we used voltage-sensitive dye (VSD) imaging to study the responses of interneurons in the local bend network. Leech interneurons respond to synaptic input from mechanoreceptors with clear changes in their membrane potential, sometimes accompanied by very small action potentials (<5 mV, Nicholls and Purves, 1970; Lockery and Kristan, 1990; Marin-Burgin et al., 2008; Pirschel et al., 2018). Recent advances in voltage-sensitive dye imaging provide a useful tool to record responses of a large number of neurons simultaneously with a decent temporal resolution in one or both sides of the ganglion (Tomina and Wagenaar, 2017). It allows the monitoring of action potentials and the detection of subthreshold membrane potential changes of only a few millivolts (Miller et al., 2012). It has been used in the leech nervous system to identify synaptically coupled neurons (Taylor et al., 2003), to study the development of synaptic connections in embryos (Marin-Burgin et al., 2008), to identify neurons involved in decision making (Briggman, 2005; Briggman and Kristan, 2006), to analyze the effects of inhibition in the local bend circuit (Baca et al., 2008), to identify the preparatory network for the movement (Frady et al., 2016), and to construct functional maps of cells involved in a set of different behaviors (Tomina and Wagenaar, 2017).

In this paper, we study the activity of the local bend network driven either by electrical stimulation of a single pressure mechanoreceptor or by natural tactile stimulation on the skin involving the activation of 4–6 mechanoreceptors. To this aim, we used VSD imaging to monitor the responses of all the visible interneurons on the ventral side of the ganglion. We focused on the question of how the local bend interneurons respond to stimuli with different intensities either provided by intracellular current injection into a single pressure mechanoreceptor or by tactile skin stimulation that activated the whole population of mechanoreceptors with overlapping receptive fields. Here we show for the first time that single mechanoreceptor stimulation elicits less pronounced network activity than natural touch stimulation. This finding supports the hypothesis of a local bend circuitry that is more complex than a simple three-layered feed-forward network.

## Methods

### Experiments

#### Animal and preparations

**Figure 1:**
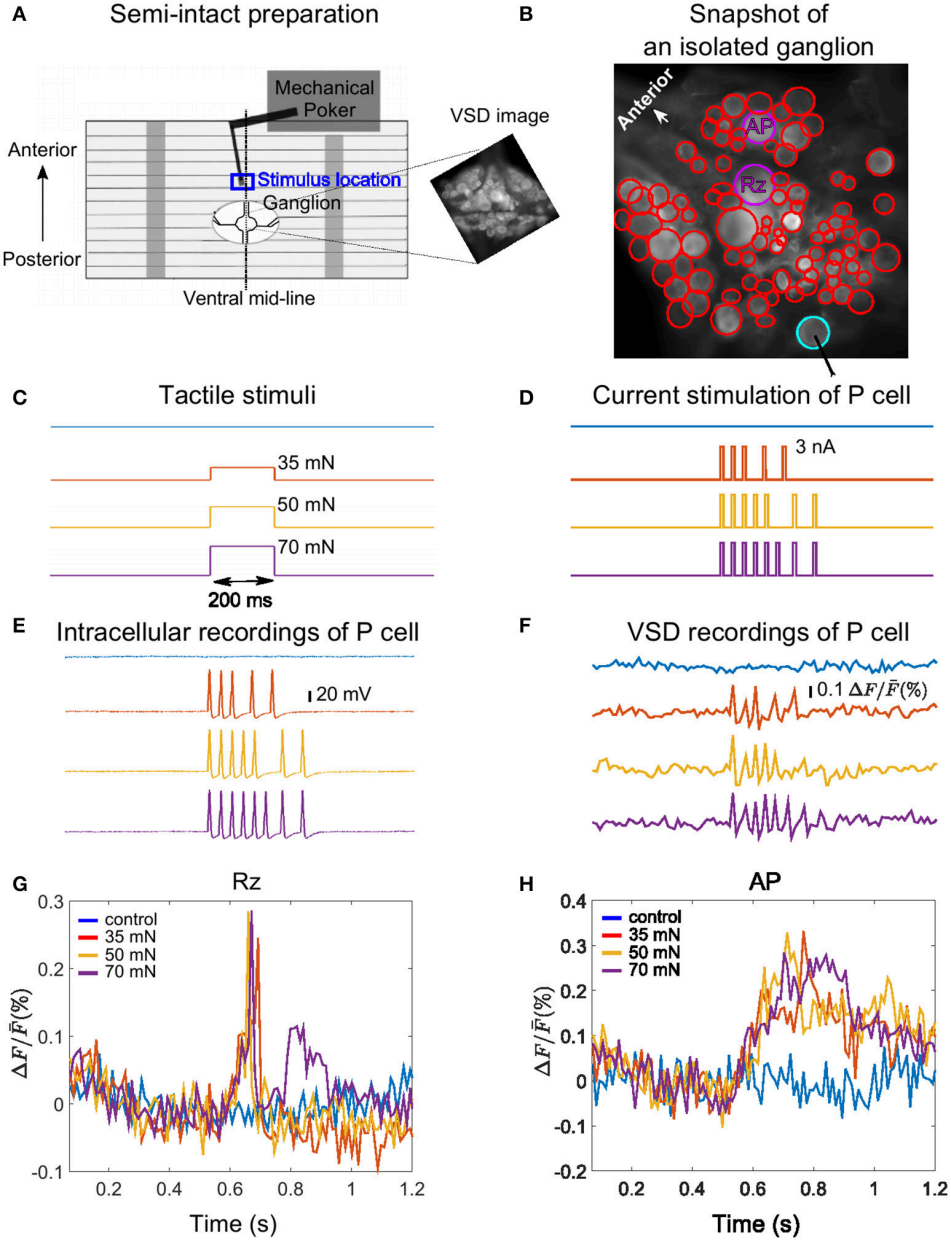
Experimental design, example VSD recording and cell responses. **(A)** Body-wall preparation of a leech ganglion (see Pirschel and Kretzberg, 2016). The skin was bisected on the dorsal mid-line of the body-wall and pinned ventral side up with a small hole allowing access to the ganglion. The tactile stimulus was presented to the 3rd annulus at the ventral mid-line (blue box), while the ganglion was imaged with VSD recordings. The right inset shows a frame of voltage-sensitive dye recording of the ganglion with the full resolution of the camera (512 × 512 pixels), covering approximately 1 × 1 mm^2^. **(B)** Snapshot image of an isolated midbody ganglion preparation taken with the full resolution of the camera (512 × 512 pixels). Circles depict regions of interest corresponding to cell bodies (red, purple and cyan). The cells were detected in two different layers of a stack, with the full resolution of the camera. The position of the stimulated P cell is indicated by the cyan circle, and the two cells for which responses are shown in panels G and H are marked by purple circles. **(C)** Tactile stimuli were applied for the duration of 200 ms with low (35 mN, red), medium (50 mN, yellow), and high (70 mN, purple) intensities. Responses were compared to control condition (blue). **(D)** Intracellular current pulses used to stimulate the P cell, mimicking responses to weak, medium and stronger intensities. The duration of each impulse was 10 ms. **(E,F)** Intracellularly evoked action potentials **(E)** and simultaneous VSD recordings **(F)** of the P cell in response to the intracellular current stimulation shown in **(D**). **(G,H)** Sample VSD signals of a Retzius cell (Rz) and an AP cell (both marked purple in **B**) show responses to spikes of the stimulated P cell (cyan in **B**, response traces in **E,F**).

Adult hermaphrodite medicinal leeches (*Hirudo verbana*), weighing 1–2 g, were obtained from Biebertaler Leech Breeding Farm (Biebertal, Germany) and maintained at room temperature in tanks with ocean sea-salt diluted with purified water (1:1,000). According to German regulations, no approval of an institutional ethics committee was required for these invertebrates. The animals were anesthetized with ice-cold saline (Muller and Scott, 1981) before dissection. All experiments were performed at room temperature. Two types of preparations were used for recordings: isolated ganglia dissected from segment 10 and reduced body-wall preparations of segment 9–11, in which the innervations of segment 10 remained intact so that natural responses of interneurons to the stimuli applied to the skin could be recorded. In the body-wall preparation, as described previously (see Pirschel and Kretzberg, 2016), the skin was bisected on the dorsal mid-line of the body-wall, and the ganglion 10 was pulled from its original location in the 3rd annulus of segment 10 to relocate to the 5th annulus, where a small hole was cut into the skin allowing access to the ganglion (Figure 1A). Preparations were pinned ventral side up in sylgard-coated dishes containing saline. The ganglion was desheathed by removing the glia sheath from the ventral side of the ganglion, package by package.

#### Tactile stimuli

We used a Dual-Mode Lever Arm System (Aurora Scientific, Model 300 B; Baca, 2005; Thomson and Kristan, 2006; Kretzberg et al., 2016) with a poker tip size of 1 mm^2^ to apply step-like pressure stimuli with different pressure intensities of 35, 50, and 70 mN for a fixed duration of 200 ms. In control condition the skin was not touched. Stimulus time courses are shown in Figure 1C. Tactile stimuli were applied at the ventral mid-line (marked with dashed line in Figure 1A) to the middle (3rd) annulus of segment 10, which was identified by the location of the sensilla (Blackshaw et al., 1982). The blue rectangle in Figure 1A depicts the approximate location of the stimulation. The VSD recordings were started 500 ms before tactile stimulation and lasted for at least 1 s.

#### Electrophysiology

We used standard intracellular recording techniques, as described in the study of Pirschel and Kretzberg (2016), to monitor and stimulate P mechanoreceptor neurons, simultaneously to VSD recordings from all visible cells on the ventral side of the ganglia. Glass electrodes with resistances between 20 and 40 MΩ, filled with potassium acetate (3 M), were used for recordings. Neuronal responses were recorded at a sample rate of 10 kHz and analyzed using custom-written MATLAB scripts (MathWorks).

P cells could be unequivocally identified by their characteristic size and position in the ganglion and their electrical properties (Nicholls and Baylor, 1968). Current stimuli (Figure 1D) consisting of 5, 7, and 8 suprathreshold step pulses (3 nA, 10 ms) were injected to mimic P cell spike trains in response to tactile skin stimulation with low (35), medium (50), and high (70 mN) pressure intensities as shown in Figure 1C. To design these P cell spike trains, we used the subset of the data from Pirschel and Kretzberg (2016) and Kretzberg et al. (2016), in which P cell responses to tactile stimulation at the ventral mid-line of leech body-wall were recorded. According to this dataset, P cells generate between 2 and 6 spikes (mean = 4, median = 5) in response to 35 mN, between 2 and 8 spikes (mean = 5, median = 7) for 50 mN and between 4 and 8 spikes (mean = 5, median = 8) for 70 mN (see Kretzberg et al., 2016, Figure 3). The median spike counts and inter-spike interval values were considered to design the electrical P cell stimulus protocol, resulting in 10 ms long stimulation with a current pulse of 3 nA at times [535, 570, 605, 668, 731] ms for low, [535, 570, 605, 640, 675, 763, 826] ms for medium, and [535, 570, 605, 640, 675, 710, 763, 826] ms for high pressure intensities. In our experiments, electrical stimulations of the P cell were separated by a time lag of 30 s, followed by a control trial without stimulation (blue traces in Figures 1D–F).

#### VSD imaging

Voltage-sensitive dye imaging technique was used to monitor the activity of all visible neurons on the ventral side of the ganglion in response to different stimulus intensity conditions. The glia sheath on the ventral side of ganglia was removed, and ganglia were stained with 200 nM VF2.1.CL dye (λmax = 522 nm, λem = 535 nm, see Miller et al., 2012). The sequence of images was obtained by a CCD camera (Photometrics QuantEM:512SC) mounted on a microscope [Zeiss Examiner.D1, objective plan-apochromat 20 x/1.0 DIC (UV)] at the rate of 94.5 frames per second and the resolution of 64 × 128 pixels. Snapshot images with a full spatial resolution of the camera, 512 × 512 pixels, were taken from different depths in stacks, based on which regions of interest (ROIs) representing individual cell bodies were drawn manually. Figure 1A (right inset) and Figure 1B depict snapshot images from ganglia in a semi-intact preparation and an isolated ganglion, respectively.

### VSD signal processing

We followed the previously developed procedure of drawing cell regions, image alignments, and normalization to extract the VSD signals from the sequence of images (Fathiazar and Kretzberg, 2015). The processing steps are explained in more details in the following sections.

#### Define cell regions

The first step of VSD signal analyses was to define the cells' regions of interests. Although the maximum spatial resolution of the camera was 512 × 512 pixels, recordings with the desired temporal frequency of almost 100 frames per second could only be achieved with a lower spatial resolution of 64 × 128 pixels. Snapshot images with the full resolution of the camera (512 × 512 pixels), taken prior or right after the VSD recordings, were only used to define the regions of interests characterizing individual cells. Due to the location of the cell bodies on the spherical surface of leech ganglia, we identified neurons in up to three snapshot images from different depths in the stack. The regions of interests from the different depths were mapped to one selected snapshot image. Figure 1B depicts regions of interests representing cell bodies with cyan, purple, and red circles, detected in two different layers of a stack in a sample VSD experiment. The resulting picture was sampled down to fit the spatial resolution of VSD recordings (64 × 128 pixels).

#### Image alignment

To extract the cells' response traces from the sequence of images, we tracked the average luminance of the pixels inside the cells' regions of interest. Since the positions of the cells might change slightly over time due to movements of cells, ganglia, or the skin preparation, the defined regions of interest might not fit the actual cells' positions on all successive frames, leading to the so-called movement artifact. To remove this artifact, all the successive frames of each recording were mapped to the sixth frame using an optical flow method (Fathiazar and Kretzberg, 2015).

The optical flow method, however, could not correct extreme cell movements especially in tactile preparations with attached body-wall. During desheating, some cells were loosed, resulting in displacement from their original location. This displacement occurred most frequently in body-wall preparations, where the ganglion was pulled slightly toward posterior, to fit the small hole cut into the skin. In this condition, the glia sheath of the ganglion was removed while some cells were under strain. Therefore, cells for which the calculated flow field indicated extreme movement were excluded from further analysis (e.g., cells marked in black in **Figures 6G**, **7B**).

#### Normalization

The absolute signal level of each trace depended on the amount of the dye bound to the cell membrane and the relative positions of the cell to the microscope lens. Cells closer to the microscope or with a higher concentration of dye appeared brighter in the images, having higher signal values. Hence, to enable comparison of the cells within or across different experiments, the signal level of each cell was normalized to its mean luminance level, which was calculated by averaging the luminance level over 30 frames before the start of the stimulation ($\Delta F/\bar{F}$).

#### Debleaching

Photo-bleaching leads to a reduced signal level over time, due to light imposed dye destruction. To estimate the effect of bleaching, we recorded a control trial without stimulation following each sequence of recordings with stimuli. For each unstimulated recording, a linear line, representing the bleaching decay, was fitted to the VSD trace of the pixels in the ROI. We then removed the bleaching artifact from the cell responses to stimulation by subtracting this fitted line to the control trial, similar to (Fathiazar and Kretzberg, 2015). The processed VSD signals of two sample cells in response to intracellular P cell stimulations are shown in Figures 1G,H.

### Detection of stimulus-activated cells using statistics-based method

The VSD signal is noisy. The lower sampling rate (94.5 frames per second) of VSD recordings compared to intracellular recordings further reduces the temporal precision of the signal. Figures 1E,F compares the intracellular recordings of a P cell with its simultaneous VSD recordings. To detect membrane potential changes of cells (<5 mV in interneurons) despite the considerable noise level, we followed the procedure explained in (Fathiazar et al., 2016). Briefly, we calculated the baseline VSD signal level for each cell by averaging the control trials without stimulation. This baseline of each cell was subtracted from the VSD recordings in stimulated trials to obtain the stimulus-induced activities. To reduce the noise level, we applied a moving average filter with the window size of three frames to the baseline subtracted signals. We then applied the statistics-based approach explained in (Fathiazar et al., 2016), to detect statistically significant deviations from the baseline at each time point. For each time frame, the number of trials were counted, in which the signal was deviated from the baseline. Stimulus-induced significant changes were detected as explained briefly in the following sections.

#### Detection of significant deviations from baseline at each time point

**Figure 2:**
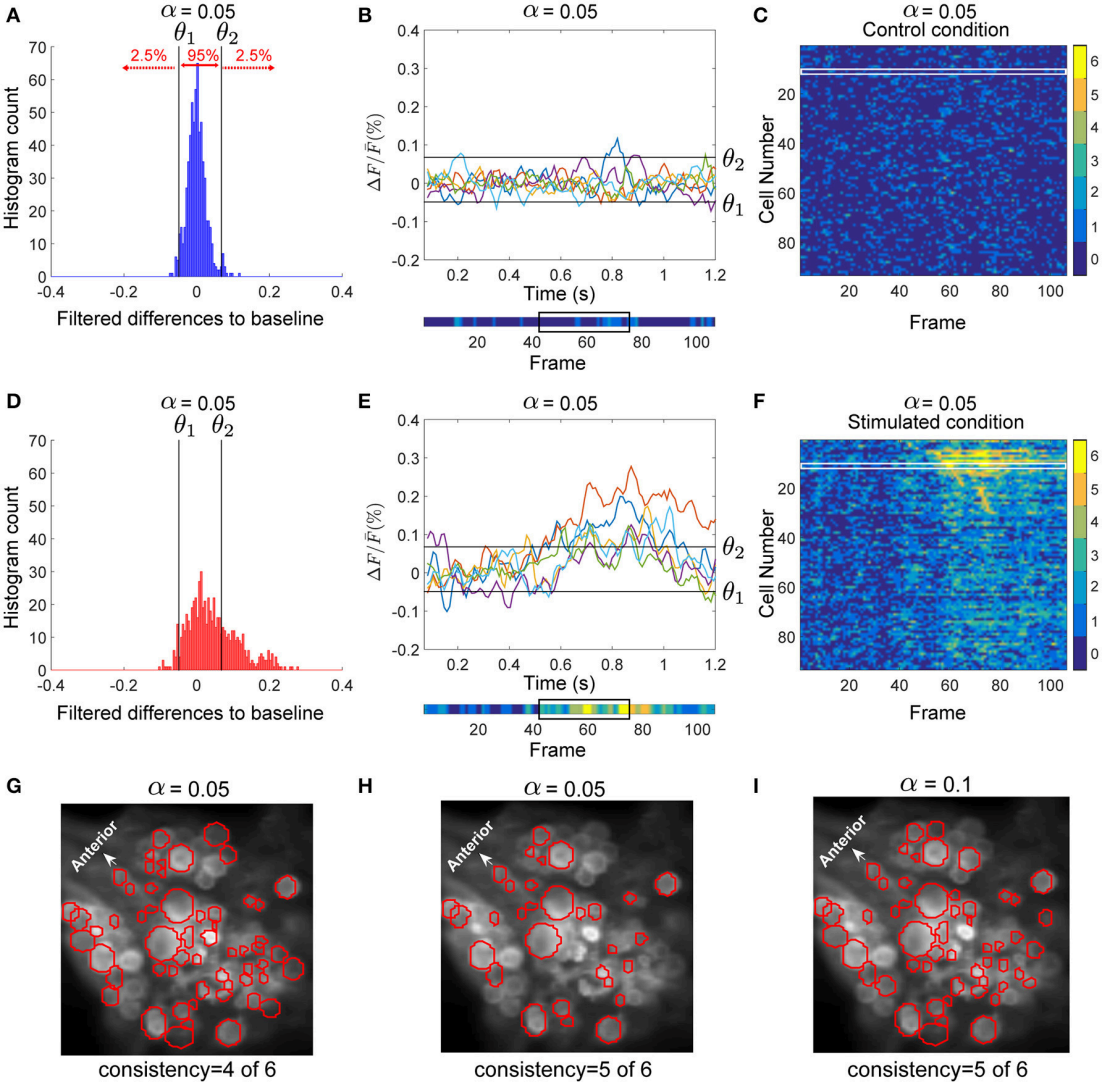
Detection of stimulus-activated cells. **(A)** Histogram of the filtered difference signals of an AP cell pooled over six trials (110 frames each) in control condition, plotted in **(B)**. Threshold values θ_1_ and θ_2_ (black vertical lines in **A**) separated the top 2.5% and bottom 2.5% (α = 0.05) values in control trials. (**B**, lower inset) The activity map shown as a colored line with values between dark blue (0) and yellow(6) (see color bar in **C,F**), indicates how many of the 6 trials (**B**, upper panel) crossed the threshold values (black horizontal lines). The black box indicates frames 43–77 (see **E**). **(C)** The activity map of all 93 selected cells in the ganglion for six trials under control condition. Each row corresponds to one cell; e.g., row 12 (in the white box) corresponds to the activity map of the AP cell shown in **(B)**, lower inset. **(D)** Histogram of the filtered difference responses of the AP cell (plotted in E) to intracellular P cell stimulation over six stimulated trials (110 frames each) with medium intensity stimuli. The time course of injected current is shown in Figure 1D (yellow line). Black vertical lines in **(D)** and black horizontal lines in **(E)** indicate thresholds calculated in control condition to determine significant deviations from the baseline in stimulated condition (see **A,B**). (**E**, upper inset) Signal values above θ_1_ and below θ_2_ indicate significantly different responses (α = 0.05). (**E**, lower inset) Activity map of the AP cell responses in stimulated condition. The time frames between the onset of the intracellular stimulus (frame 43), and the offset of the stimulus plus five frames (frame 77) are indicated with a black box. **(F)** Activity map of 93 selected cells in the ganglion for six trials (110 frames each) under stimulated condition. Cells were sorted according to their sequence of activation after stimulus onset. Compared to the control condition **(C)** many cells showed more significant activities in response to P cell stimulation. From these activity maps, individual cells were classified as stimulus activated, if they responded consistently at least in one frame between the onset of the intracellular stimulus (frame 43), and the offset of the stimulus plus five frames (frame 77). **(G)** Cells in red circles were detected as stimulus activated with the consistency value of 4, meaning that these cells had at least one dark green (4), orange (5), or yellow (6) pixel in the above-mentioned period of the activity map. **(H)** Stimulus activated cells with the consistency value of 5 are shown in red; i.e., the cells with at least one pixel in orange (5) or yellow (6) induced by P cell stimulation. **(I)** Cells in red were detected with the significance level of α = 0.1 and consistency criteria of 5. The significance level of 0.1 implies that threshold values, θ_1_ and θ_2_, were calculated to separate the 5% highest and lowest values, respectively, resulting in a large number of neurons classified as stimulus activated than for α = 0.05.

For each cell, the data from preprocessed control trials were pooled. Figure 2A shows the histogram of the deviations of all control trials from baseline calculated for the sample AP cell. We calculated the threshold values separating the 2.5% of the lowest values (θ_1_) and 2.5% of the highest values (θ_2_) of the pooled deviations from baseline in the control trials (black lines in Figures 2A,B). These thresholds were applied to detect significant deviations from the baseline of the stimulated trials (Figures 2D,E). For each time point, the deviation from baseline was compared to the thresholds θ_1_ and θ_2_. If any of these thresholds was crossed, the data point was classified as a significantly high or low value.

#### Activity map

To distinguish the cells' activity from the noisy background, we counted frame-by-frame the number of trials showing significant deviations from the baseline as a measure of consistently changing activity over repeated trials. The resulting numbers for each time frame and each cell were combined into an activity map. The lower insets of Figures 2B,E depict the activity map for one sample AP cell in control and stimulated conditions, respectively, corresponding to the 6 trials shown above each inset. The color of each pixel in the activity map [between 0 (blue) and 6 (yellow)] indicates the number of significant deviations from the baseline. Figure 2F shows the activity map (*I*(*j, k*)) of 6 stimulated trials for all recorded cells and Figure 2C for control trials. Each row (*j ϵ* {1, …, 93}) corresponds to an individual cell, while the columns (*k ϵ* {1, …, 110}) are the frame numbers. The frame numbers, *k ϵ* {1, …, 110} correspond to the sample points in the range of 0.07 < *t* < 1.2 s. *I*(*j, k*) ϵ {0, …, 6} shows in how many of 6 trials the cell *j* at frame *k* was active. From these activity maps, individual cells were classified as ‘stimulus-activated’ if the summed value of at least one frame between the onset of the impulse stimulus (sample point *t* = 0.5 s, frame 43), and offset of the stimulus plus 5 sample points (for P cell stimulation with medium intensity, *t* = 0.88 s, frame 77, see black boxes in Figures 2B,E, lower inset) was equal to or exceeded the criteria value of 5 out of 6. Apparently lower consistency values or larger significance levels lead to a larger number of cells classified as stimulus-activated cells. Figures 2G–I compares the stimulus-activated cells (in red) found for consistency criteria of 4 and 5 and for significance levels of 0.05 and 0.1. In this paper we used the relatively strict values of a consistency criterion of 5 out of 6 trials and significance level of 0.05. These values provide a conservative estimation of stimulus-activated cells by minimizing the number of false positives.

### Detection of stimulus-activated cells using friedman's significance test

As an alternative method to identify stimulus-activated cells we applied Friedman's test (Hollander et al., 2013; *p* < 0.001) to find the cells responding significantly different to stimulated conditions compared to control condition. The test is an alternative measurement to repeated ANOVA, but using ranks rather than the original data values. In this test, the difference to baseline VSD values calculated for each stimulus conditions were ranked separately for each cell. Then, ranks obtained for all cells were grouped according to the stimulus condition they were elicited by. The null hypothesis was that the distributions of ranks were identical for control and examined stimulus condition. If the null hypothesis was rejected, response ranks of the examined stimulus condition were judged to differ significantly from the rank distributions obtained for the control condition, showing a significant effect of the stimulation on the response of the recorded cells.

### Detection of significance differences between stimulus conditions using friedman's significance test

For cells identified as stimulus-activated, significant differences in neuronal responses to different stimulus intensity conditions (including control condition) were tested with the Friedman's test (Hollander et al., 2013; *p* < 0.001), described in more details in the study of Pirschel and Kretzberg (2016). As before ranks obtained for all cells were grouped according to the stimulus condition they were elicited by. Here, the null hypothesis was that the distributions of ranks were identical for all stimuli. If the null hypothesis was rejected, response ranks of at least one stimulus condition were judged to differ significantly from the rank distributions obtained for the other stimulus values, showing a significant effect of the stimulation on the response of the stimulus-activated cells.

Individual cell responses to different stimulus conditions were compared by calculating the average difference to baseline VSD values ($\bar{VSD}$). The averaging was performed on the time window used to detect the cells' significant activation (stimulus onset till offset plus 5 frames). The cell's response was labeled according to comparison of its responses to different stimulus conditions as “increase with stimulus intensity” if ${\bar{VSD}}_{low\text{\_}stim}<\;{\bar{VSD}}_{medium\text{\_}stim}<\;{\bar{VSD}}_{high\text{\_}stim}$ and “decrease with stimulus intensity” if ${\bar{VSD}}_{low\text{\_}stim}>\;{\bar{VSD}}_{medium\text{\_}stim}>\;{\bar{VSD}}_{high\text{\_}stim}$. Otherwise the cell's response was labeled as “change non-monotonic with stimulus intensity”.

We then applied a multiple comparison test with *post-hoc* correction (function multcompare, MATLAB statistics toolbox) to the ANOVA table obtained by Friedman's test to determine pairwise significant differences. The MATLAB default type of critical value (Tukey-Kramer; Milliken and Johnson, 2009) with significance level of 0.05 was used to test significant differences between pairs of VSD responses. The pairwise significant differences were calculated between combinational pairs of control conditions and stimulated conditions (pairs of 0 vs. 35 mN | 0 vs. 50 mN | 0 vs. 70 mN) and also between combinational pairs of the stimulated conditions (pairs of 35 vs. 50 mN | 35 vs. 70 mN | 50 vs. 70 mN).

## Results

### VSD signals reflect membrane potential responses to stimulation

**Figure 3:**
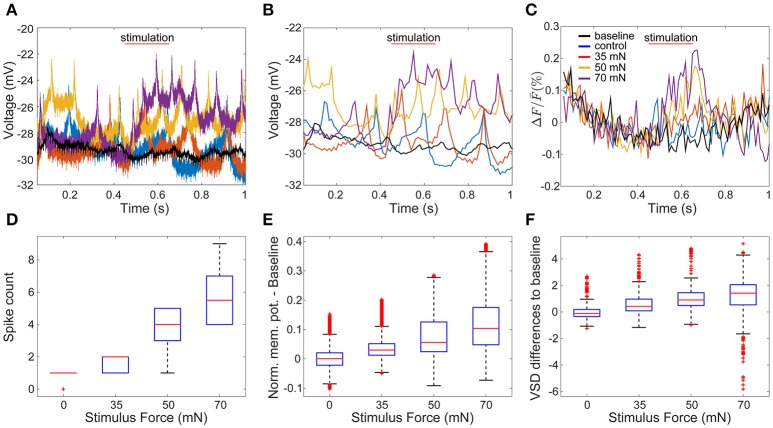
Comparison of intracellularly recorded membrane potential and VSD signal of the same cell. **(A)** The membrane potential of an AP cell was recorded intracellularly simultaneously to VSD recordings **(C)**. In **(A**–**C)** red, yellow and purple lines are recorded example responses to tactile stimulation with low, medium and high intensities in semi-intact preparation (as shown in Figure 1C). Blue lines show example responses to control condition. Black lines indicate baseline, calculated as the average of all traces recorded during control condition. **(B)** Temporally down sampled version of the membrane potential shown in **(A)**, according to the camera sampling rate. **(D–F)** Boxplots of response features obtained for 14 trials in each of four stimulus conditions: **(D)** intracellularly detected spike counts between stimulus onset and stimulus offset plus five frames, **(E)** intracellularly recorded membrane potential differences to baseline, and **(F)** VSD signal differences to baseline.

To evaluate how accurately VSD signals reflect membrane potential changes, we compared the VSD signals of one sample cell with its corresponding intracellular membrane potentials. We recorded the response of an AP cell intracellularly while imaging the whole ganglion with VSD in a body-wall preparation. The AP cells have relatively big somata compared to their neighboring cells and therefore could be identified reliably across preparations. Figures 3A,C depict intracellular recordings from the AP cell and its simultaneously recorded VSD signals in response to different stimulus conditions (as shown in Figure 1C) of low (35 mN), medium (50 mN), and high (70 mN) pressure intensities as well as control condition (0 mN). To allow direct visual comparison of both types of recordings, the intracellularly recorded membrane potential (Figure 3A) was resampled based on the camera exposure-readout paradigm (Figure 3B). The VSD signal levels (Figure 3C) increased similarly to the intracellularly recorded graded membrane potential changes (Figures 3A,B). For the statistical comparison of the VSD signal with the intracellularly recorded membrane potential, similar preprocessing steps were applied to both types of data. The baseline was calculated by averaging over the control trials and subtracted from the normalized responses. Spike counts were calculated based on the intracellular recordings at the same time interval used to detect the stimulus-activated cells (see Method section Activity Map). Stimulus intensity showed a significant influence (*p* < 0.001, Friedman's test) on VSD differences from baseline (Figure 3F), as well as on intracellularly measured membrane potential differences from baseline (Figure 3E) and spike counts (Figure 3D). For all of these three response features, significant differences (α < 0.05, multiple comparison test) were found between any of the stimulated conditions and the control condition (Figures 3D–F). Moreover, for the intracellularly recorded graded membrane potential changes (Figure 3E) and spike counts (Figure 3D), significant differences (α < 0.05, multiple comparison test) were also found between all combinational pairs of stimulated conditions, e.g., between medium and strong intensity conditions; while the VSD signals (Figure 3F) only showed significant differences between weak and strong stimulation. Since the low signal-to-noise ratio of the VSD recordings did not allow the reliable estimation of spike counts based on the optical signals, all further analyses were performed based on the graded membrane potential changes.

### Interneurons' responses depend weakly on the intensity of intracellular stimulation of a single P cell

To study the question of how interneurons process the tactile stimulus information provided through mechanosensory neurons, we examined in the first step how a single sensory neuron influences the network activity. We recorded from all visible cells on the ventral side of the ganglion with voltage-sensitive dye imaging during simultaneous intracellular current stimulation of a single P cell. The intracellularly injected current stimuli consisted of sequences of current pulses, designed to mimic P cell spike trains in response to tactile stimulations with four different pressure intensities: control (0 mN), low (35 mN), medium (50 mN), and high (70 mN) pressure intensities (Figure 1D).

Our data was collected in 6 different experiments, with at least 24 trials in each experiment, including 6 trials of each stimulus conditions of control (0 mN), low (35 mN), medium (50 mN), and high (70 mN) pressure intensities mimicked by intracellularly injected current. On average 114 (max: 136, min: 93) of ~160 cells on the ventral side of a leech ganglion were visible clearly enough to allow VSD data analyses. An example experiment with 93 analyzed cells is shown in Figure 1B (red, cyan, and purple circles). The sample VSD traces of two relatively large and reliably identifiable cells (one Retzius cell and one AP cell, marked in purple in Figure 1B) in response to intracellular stimulations of a P cell (cyan in Figure 1B, response traces in Figures 1E,F) are shown in Figures 1G,H.

**Figure 4:**
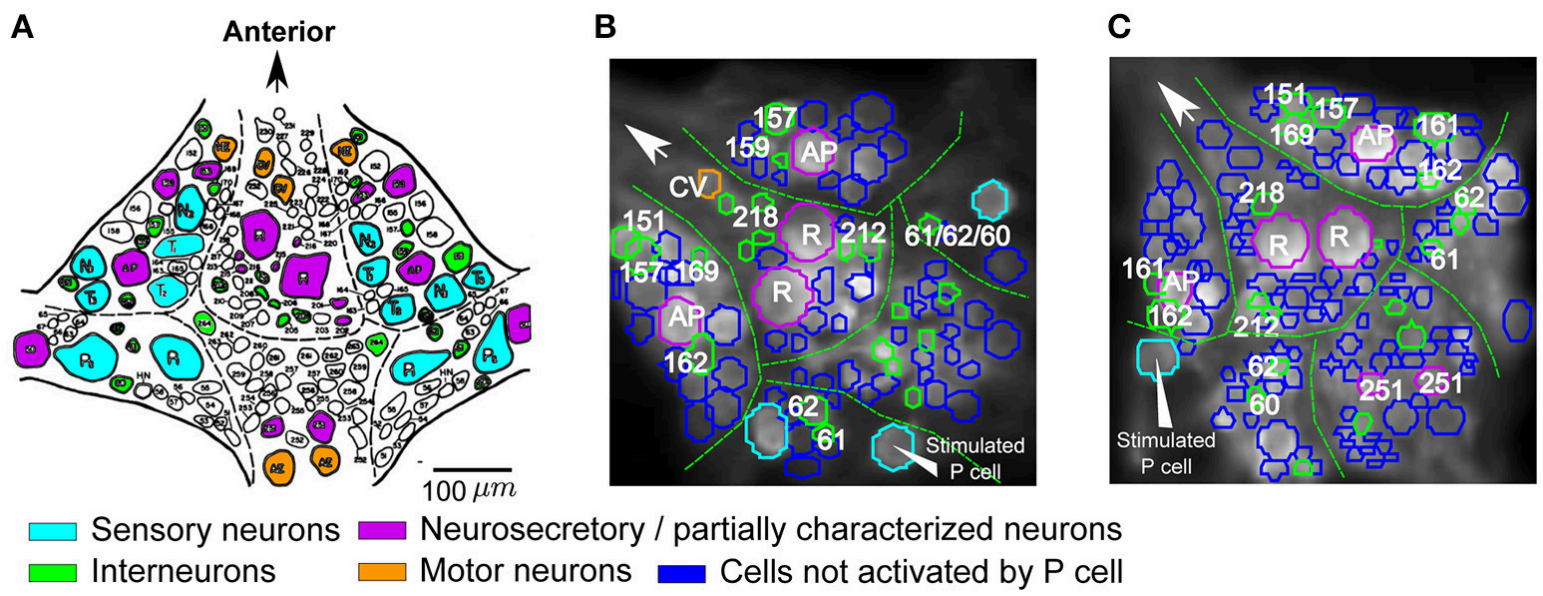
Cells responding to single P cell activity. **(A)** Standard ganglion map with cells colored based on their published functions (based on Wagenaar, 2015): cyan, sensory neurons; orange, motor neurons; green, postsynaptic interneurons to P cells; purple, neurosecretory and partially characterized neurons. Scale bar: ~100 μm. **(B,C)** Two example VSD preparations showing cells activated by P cell stimulation with medium intensity, colored according to their putative functions (see **A**). The stimulated P cells are indicated by triangle arrows. Activation was detected with a threshold value of α = 0.05 and consistency criterion of 5 out of 6. Cells were labeled based on their location and size in comparison to the standard ganglion map (A).

Six trials of each intensity condition were used in each of the six experiments to identify stimulus-activated neurons using the statistics-based method (section Detection of Stimulus-Activated Cells Using Statistics-Based Method) on the one hand and the Friedman's test (section Detection of Stimulus-Activated Cells Using Friedman's Significance Test) on the other hand (see Table 1). For the statistics-based method the relatively strict parameters of the threshold value (α = 0.05) and the consistency criterion (5 out of 6 trials) led to detection of significant response deviations from the baseline activity in approximately 1/3 of all analyzed cells for all stimulus conditions. Figures 4B,C show two example experiments with 30 and 28 cells in green, cyan, orange, and purple, which were identified as stimulus-activated by electrical P cell stimulation mimicking a tactile stimulus with medium intensity. By increasing the threshold value and/or decreasing the consistency criterion, the number of cells identified as stimulus-activated cells would increase (for a detailed description see section Detection of Stimulus-Activated Cells Using Statistics-Based Method and Figure 2). Friedman's test (*p* < 0.001) identified slightly more cells (Table 1) and reconfirmed that similar numbers of cells responded significantly across stimulus conditions.

**Table 1:** Percentage of stimulus-activated cells driven by electrical stimulation of a single P cell.

	**Number of stimulus-activated cells (%)**		
	**35 mN**	**50 mN**	**70 mN**
Criterion: 5 of 6	33	38	37.5
Friedman's test	44	46	45
Friedman's test AND Criterion: 5 of 6	20	25	23

*The average percentage of the stimulus-activated cells were calculated over six experiments in response to different stimulus conditions of low (35 mN), medium (50 mN), and high (70 mN) pressure intensities mimicked by intracellularly injected current. The cells were detected as stimulus-activated using: statistics-based method with consistency criterion of 5 out of 6 trials (section Detection of Stimulus-Activated Cells Using Statistics-Based Method, α = 0.05), Friedman's test (section Detection of Stimulus-Activated Cells Using Friedman's Significance Test, p < 0.001) and the combination of statistics-based method and Friedman's test*.

Based on the locations and relative sizes of the cells, some of the cells were identified by comparing VSD images of the ganglia (Figures 4B,C) with the standard ganglion map (Figure 4A). The cells that were classified as stimulus-activated by our statistics-based method matched well with the literature. In particular, we found both Retzius cells and AP cells with relatively big cell sizes, cells 157, 159, 161, 162, 169, 212, 218 which were reported as local bend interneurons by Lockery and Kristan (1990), and other neurons with published synaptic inputs from P cells such as interneurons 60, 61, 62 (Kristan et al., 2005; Kretzberg et al., 2016), cell 151 (a premotor neuron; Marín-Burgin and Szczupak, 2000), and cell 251 (partially characterized neuron; Frady et al., 2016). The remaining P-activated cells comprised putatively cells 204, 205, 208 reported previously as P-activated neurons (Kristan et al., 2005) and cells 261, 262, and 255 (premotor neurons; Fan et al., 2005) whose responses to mechanoreceptor inputs were not reported before.

**Figure 5:**
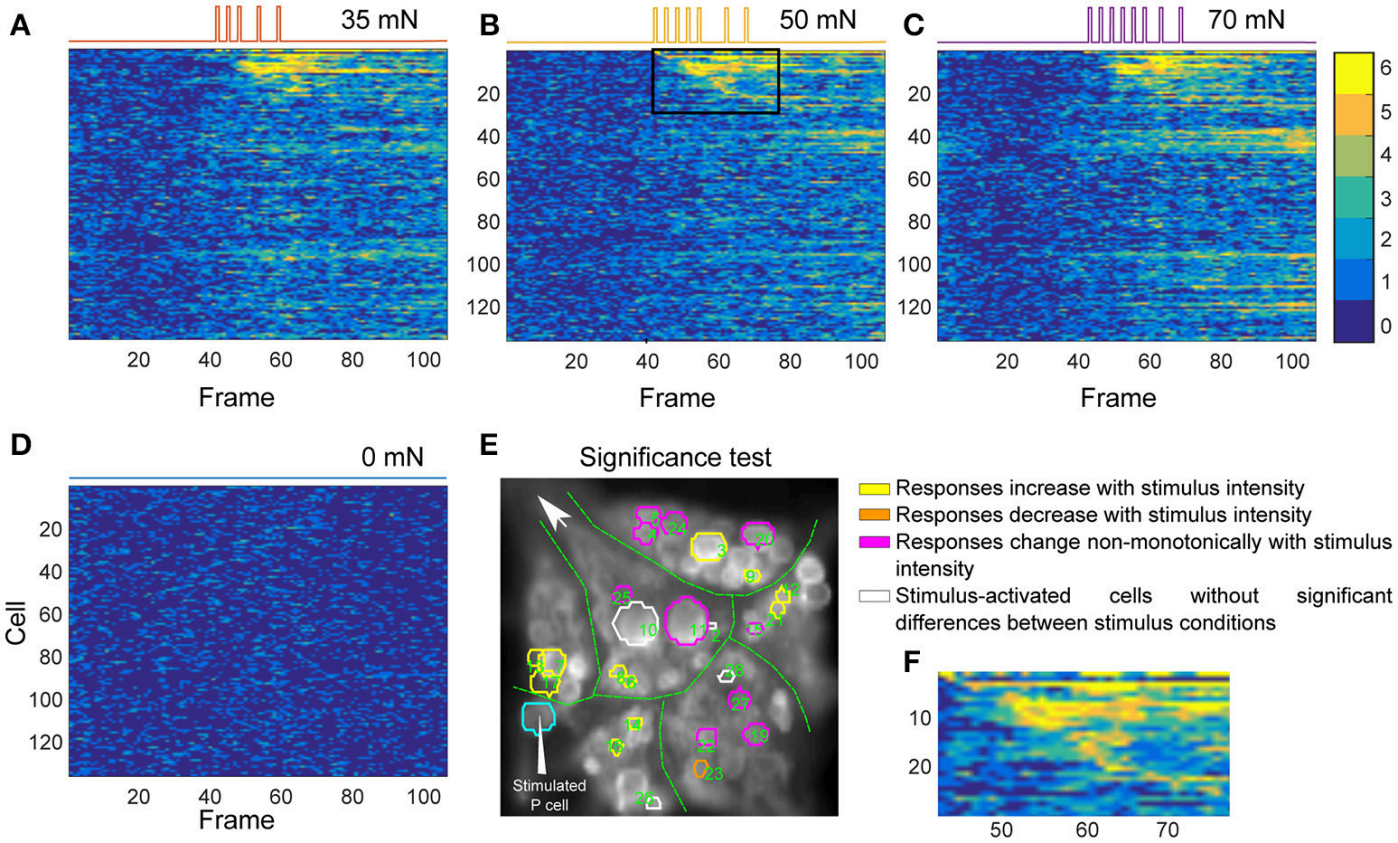
Influence of P cell spiking patterns on postsynaptic neurons. **(A–D)** Activity maps of all cells in the ganglion **(E)** for each condition of intracellular P cell current stimulation, indicated at the top of each panel, corresponding to different stimulus intensity conditions introduced in Figure 1D sorted according to cells' response onsets to medium intensity stimulation. Colors (see color bar ranging from 0 to 6) of each pixel indicate the number of trials, for which the activity of a specific cell (row) significantly differed from baseline at a specific time frame (column). Cells were numbered according to their sequence of activation after onset of stimulation with medium intensity. Locations of the cells in the first 28 rows are indicated by the green numbers in **(E)**. **(E)** Response classes of stimulus-activated cells in the same preparation as in Figure 4C are indicated by different colors according to their response patterns across stimuli. Numbers in E correspond to the cell numbers in the activity map of panel **(A–D)**. Cyan: Intracellularly stimulated P cell. Yellow: Stimulus-activated cells with significantly different (Friedman's test, *p* < 0.001) responses to different stimulus conditions with mean VSD signal consistently increasing with increasing stimulus intensity. Orange: Stimulus-activated cell with significantly different responses to different stimulus conditions with mean VSD signal consistently decreasing with increasing stimulus intensity. Magenta: Stimulus-activated cells with significantly different responses to different stimulus conditions but without monotonic dependency of VSD signal on stimulus intensity. White: Stimulus-activated cells whose responses to different stimuli were not significantly different. **(F)** The zoomed-in view of the rectangular window, corresponding to rows 1–29 and columns 43–78 of the activity map in **(B)**.

Figures 5A–D depict the activity maps of all cells in Figure 4C to three stimulus conditions and the control condition. Each row displays the results for one of the analyzed 136 cells over 110 recorded time frames (x-axis). Each pixel indicates the number of significant deviations from the baseline over 6 trials with colors ranging from dark blue (0–no significant activation) to yellow (6–significant activation in all trials). For example the pixel in row 6 and column 60 with the color yellow indicates that the cell number 6 deviated significantly from baseline in all of six trials at the time frame 60. The rows of the activity maps in Figures 5A–D were sorted by the timing of their first significant deviation from the baseline in at least 5 out of 6 trials in medium intensity condition (Figure 5B). Figure 5F shows a blow-up from Figure 5B (black box) of the activity map of the 28 stimulus-activated cells indicated in Figure 4C. Note that the numbers in Figure 5E do not correspond to the standard ganglion map in Figure 4A, but to the sequence of their activation after stimulus onsets according to the row numbers in Figures 5B,F.

The activity maps obtained for different stimulated conditions resemble each other strongly (Figures 5A–C), matching the observation of very comparable numbers of stimulus-activated cells found for different stimulus intensities (Table 1). These similarities are probably caused by the comparable spiking patterns of P cells induced by our stimulation mimiking P cell responses to different touch pressure intensities (five impulses for low intensity, seven for medium and eight for higher intensity condition). Moreover, due to the low signal-to-noise ratio of VSD signals, the subtle differences in postsynaptic responses might not show up clearly. However, despite the visual similarity of activity maps obtained for the three stimulated conditions, calculating the number of significant activations in at least 5 of 6 trials (yellow and orange pixels) revealed that the activity of cells increased with increasing simulated touch pressure intensities used for intracellular P cell stimulation. For the stimulus-activated cells (first 28 rows in Figures 5A–C) total numbers of 272, 291, and 305 time frames were found to be significantly different from the baseline in at least 5 of 6 trials for the three increasing stimulus pressure intensities. In contrast, in control condition none of the time frames met this criterion for any of the first 28 cells (Figure 5D).

The influence of different stimulus intensity conditions on neuronal responses of stimulus-activated cells was further tested with a multiple comparison pairwise test (see section Detection of Significance Differences between Stimulus Conditions Using Friedman's Significance Test for detailed description). Most of the cells identified as stimulus-activated responded significantly different between at least one pair of stimulated conditions and control using multiple comparison test. In the example experiment shown in Figures 4C and 5, 23 of the 28 stimulus-activated cells elicited significantly different VSD responses between stimulus conditions (Figure 5E, yellow, orange, and magenta) according to Friedman's test (*p* < 0.001). From these 23 cells with significantly different responses, 16 cells showed pairwise significant responses between stimulated and control conditions in the multiple comparison test, but none of these cells had significantly different responses between any combinational pair of stimulated conditions. Nevertheless, the average VSD signal change during stimulation was found to increase progressively in 11 cells (yellow in Figure 5E) and decreased in 1 cell (orange in Figure 5E), with increasing stimulus intensities. However, some of the stimulus-activated cells (magenta in Figure 5E) did not show any consistent increase or decrease in average VSD signals, despite their significantly different responses to different stimulus conditions.

### Interneurons' responses depend strongly on the intensity of tactile stimulation

After characterizing the effect of a single sensory cell on the network activity, tactile stimuli were applied to the skin with control (0 mN), low (35 mN), medium (50 mN), and high (70 mN) pressure intensities, lasting for 200 ms (Figure 1C). Local tactile stimulation at the ventral mid-line was shown before to elicit spike responses in at least 4 mechanoreceptors (2 T and 2 P cells, Pirschel and Kretzberg, 2016). These tactile skin stimulations evoked similar or slightly weaker P cell responses as were evoked by the electrical current injections used in section Interneurons' Responses Depend Weakly on the Intensity of Intracellular Stimulation of a Single P cell. For example, stimulating the skin with an intensity of 50 mN evoked 7 spikes or less in each of the identified P cells.

We performed the tactile experiment in four different preparations, with at least 24 trials in each experiment (6 trials for each stimulus and control condition). On average 106 (max: 119, min: 95) neurons were visible enough for data analyses. To determine interneurons involved in tactile processing, the same statistical method as before (again with significance level of α = 0.05 and consistent activation criterion of 5 out of 6 trials) was applied to the cells' VSD responses. In contrast to the electrical stimulation of a single P cell, the percentages of stimulus-activated cells depended strongly on stimulus intensity, ranging from 40% for 35 mN to 71% for 70 mN (Table 2). The increase in the percentage of stimulus-activated cells with increasing stimulus intensity was confirmed by the classification based on the Friedman's test (Table 2).

**Table 2:** Percentage of stimulus-activated cells driven by tactile skin stimulation.

	**Number of stimulus-activated cells (%)**		
	**35 mN**	**50 mN**	**70 mN**
Criterion: 5 of 6	40	58	71
Friedman's test	49	56	59
Friedman's test AND Criterion: 5 of 6	30	42	50

*The average percentage of the stimulus-activated cells were calculated over four experiments in response to tactile skin stimulation with different pressure intensities of low (35 mN), medium (50 mN), and high (70 mN). The cells were detected as stimulus-activated using: statistics-based method with consistency criterion of 5 out of 6 trials (section Detection of Stimulus-Activated Cells Using Statistics-Based Method, α = 0.05), Friedman's test (section Detection of Stimulus-Activated Cells Using Friedman's Significance Test, p < 0.001) and the combination of statistics-based method and Friedman's test*.

**Figure 6:**
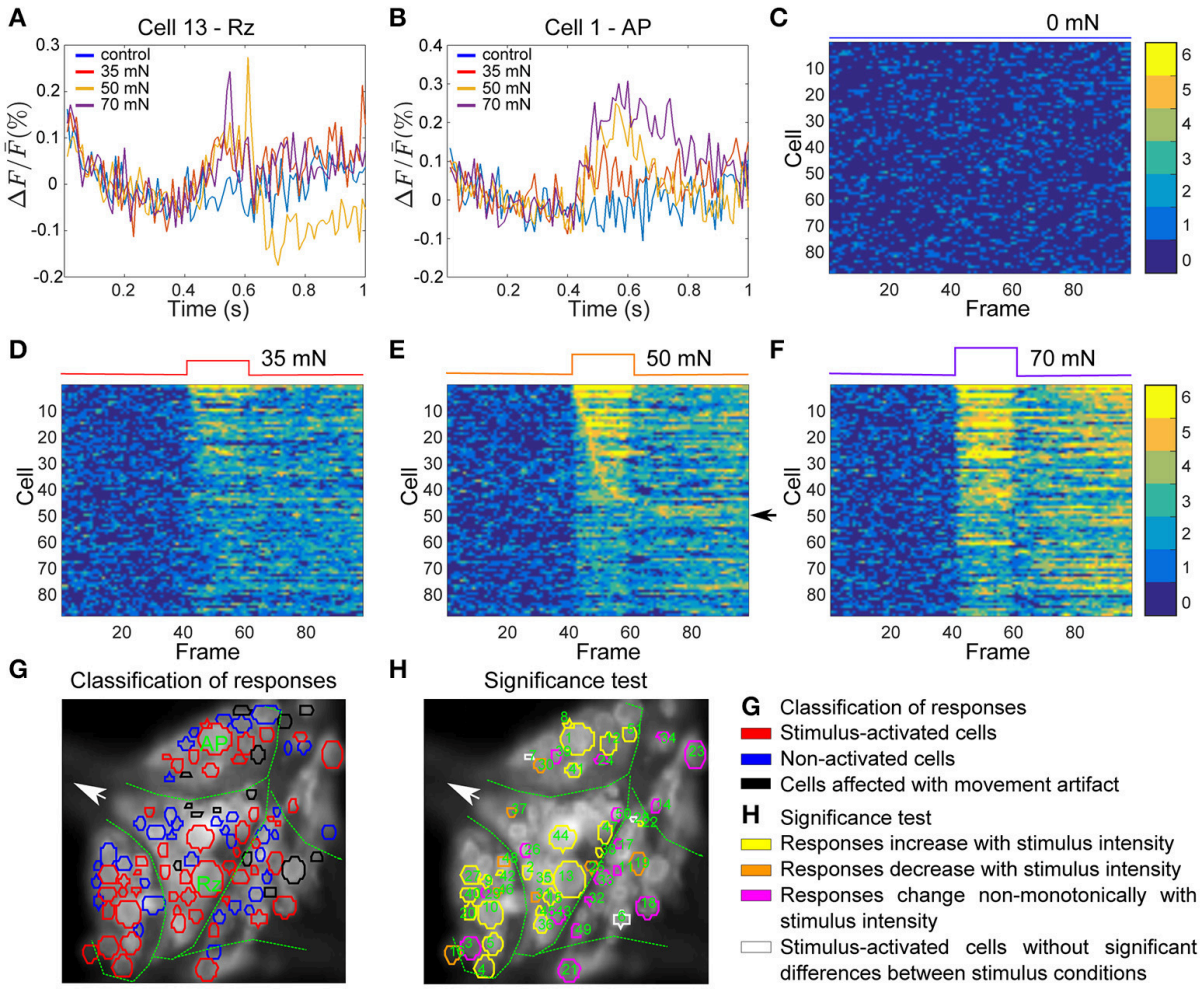
Interneurons involved in tactile processing and influence of stimulus intensity on their activities. **(A,B)** Responses of two sample cells, a Retzius cell and an AP cell (marked in **G**) to step-like pressure stimuli with different intensities of 0, 35, 50, and 70 mN for a fixed duration of 200 ms indicated in the upper panels of **(C–F)** (see Figure 1C). **(C–F)** Activity maps of all cells in the ganglion (red and blue in **G**) for each condition of tactile stimulation, indicated at the top of each panel. **(G)** Stimulus activated cells (red) were detected from all visible cells in the ganglion (blue and red) by their responses to medium intensity condition (50 mN, **E**). The threshold value of α = 0.05 and consistency criterion of 5 out of 6 were applied for activity detection. Cells indicated in black were subject to extreme movements and were excluded from further analyses. **(H)** Response classes of stimulus-activated cells are indicated by different colors according to their response patterns across stimuli. Numbers in **(H)** correspond to the cell numbers in the first 51 rows of activity maps in panel **(C–F)**. Yellow: Stimulus-activated cells with significantly different (Friedman's test, *p* < 0.001) responses to different stimulus conditions with mean VSD signal consistently increasing with increasing stimulus intensity. Orange: Stimulus-activated cells with significantly different responses to different stimulus conditions with mean VSD signal consistently decreasing with increasing stimulus intensity. Magenta: Stimulus-activated cells with significantly different responses to different stimulus conditions but without consistent dependency of VSD signal on stimulus strength. White: Cells which were classified as stimulus-activated, but which did not show significantly different responses between stimulus conditions.

Figure 6G depicts an example VSD image of a tactile stimulation experiment. The activity of 102 of ~160 neurons on the ventral surface of the ganglion were analyzed in this preparation, from which 14 neurons (shown in black) were detected to move from their position across successive frames (extreme movement artifact) and therefore were excluded from further analyses. From the remaining 88 cells (in red and blue), responses of 50 cells to the medium intensity (in red) were classified as stimulus-activated by the statistics-based method.

To further analyze the influence of stimulus intensity on neuronal responses, we calculated the activity map for each condition of control (0 mN), low (35 mN), medium (50 mN), and high (70 mN) pressure intensities (Figures 6C–F). Like before, each row denotes the activity trace of one cell, sorted by their activation in response to medium intensity stimulation, and the yellow pixels depict consistent significant activation of the cells in all six trials. The activity maps reveal that some cells responded within one or two frames directly after stimulus onset, indicating that they received direct mechanoreceptor input. In other cells consistent responses started considerably later, suggesting an indirect response to mechanoreceptor spikes caused by network activation. The activity of many cells was increased compared to baseline for the entire stimulus duration (200 ms) and even after stimulus offset. Some cells, e.g., the cells with numbers 45–51 in the activity map (indicated with an arrow in Figure 6E) seemed to respond to stimulus offset rather than onset.

Increasing the pressure stimulus intensity increased the duration and/or the amplitude of the cells' responses. Comparison of activity maps of different pressure intensities demonstrated that the number of significant deviation from baseline (number of orange and yellow pixels) increased with increasing stimulus intensity. In addition to more cells becoming active (Table 2), many cells responded stronger to higher pressure intensities, e.g., the two example cells displayed in Figures 6A,B, for which the VSD signal increased clearly with increasing stimulus pressure intensity. The duration of the network response increased, e.g., more cells continued to respond after the offset of the stimulus in high pressure intensity (70 mN) condition than in medium (50 mN) and low intensity (35 mN) conditions.

On average, different stimulus pressure intensities evoked significantly different responses (Friedman's test, *p* < 0.001) in 54% of the analyzed cells in 4 different preparations. These cells could putatively be involved in touch intensity discrimination tasks. In Figure 6H, 48 neurons (colored in yellow, orange and magenta) produced significantly different responses to different conditions according to Friedman's test. In 23 of these cells (yellow in Figure 6H) the mean VSD response values progressively increased and in 7 of these cells (orange in Figure 6H) the mean VSD response values progressively decreased with increasing tactile stimulus intensity. Applying the multiple comparison test to the 48 neurons colored in yellow, orange, or magenta in Figure 6H revealed 27 cells producing significantly different responses between pairs of stimulus condition, and 7 of them also showing significantly different responses between at least one pair of stimulated conditions, e.g., between weak and strong stimulation.

### Tactile stimulation elicits stronger network activity than intracellular stimulation of a single P cell

Comparing the results from the two previous sections, we found that tactile skin stimulation induces higher network activity than electrical stimulation of a single P cell. In fact, even the lowest touch intensity activates considerably more cells than any stimulus intensity mimicked by an electrical induced spike train in a single P cell (compare Tables 1, 2). To allow a direct comparison of the interneurons activated by electrical single P cell stimulation on the one hand and tactile skin stimulation on the other hand, both types of stimulation were applied to the same preparation as shown in Figure 6. Due to the dye phototoxicity, we had to restrict our comparison to one intensity condition, the medium intensity of 50 mN.

**Figure 7:**
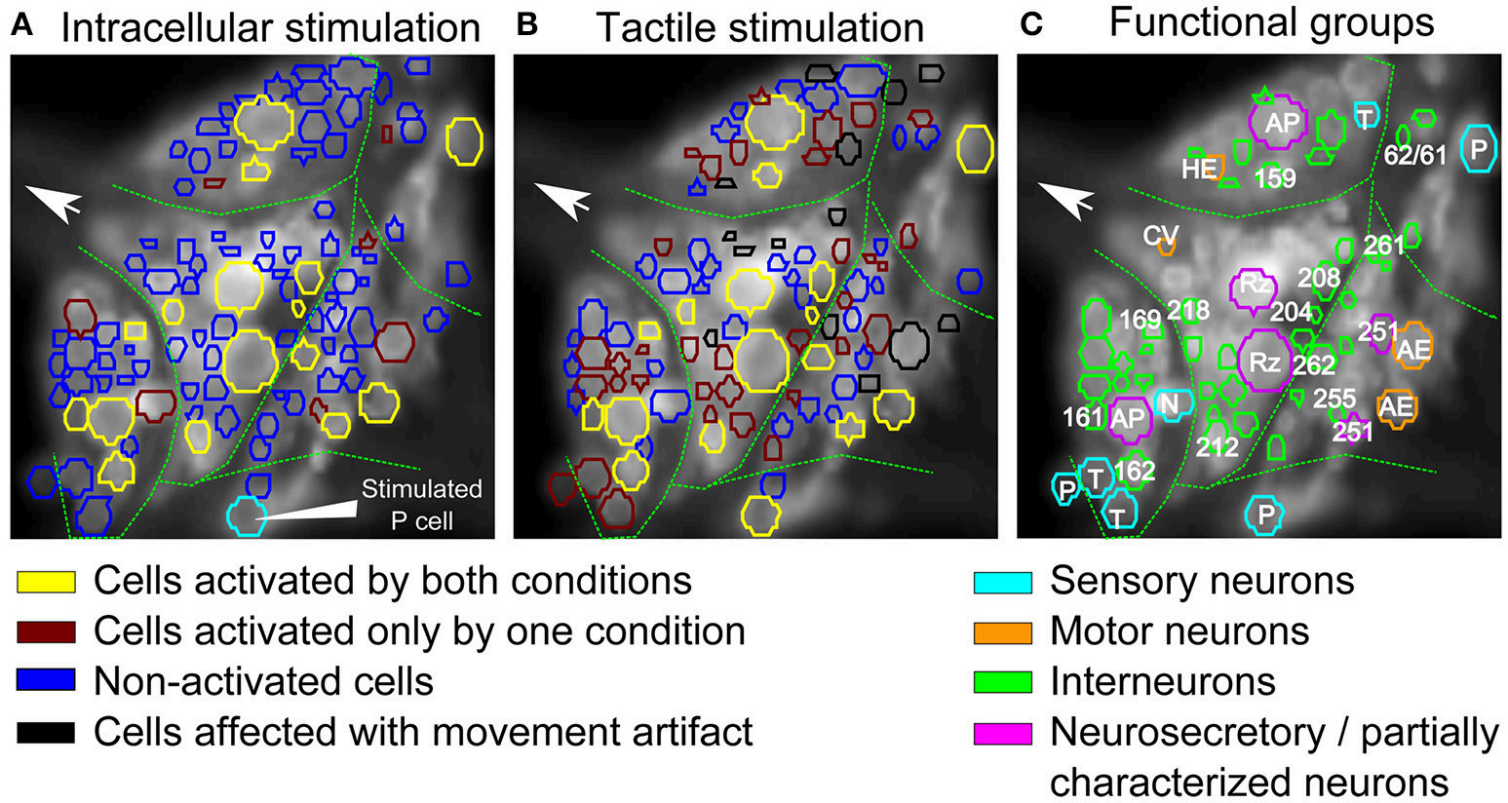
Comparison of interneurons involved in tactile processing to postsynaptic P cell interneurons. **(A,B)** Stimulus activated cells (yellow, brown) were detected from all visible cells in response to **(A)** P cell stimulation and **(B)** tactile stimulation. Cells activated by both types of stimuli are shown in yellow, cells responding to only one specific type of stimulation are shown in brown (**A**: only P cell stimulation, **B**: only tactile stimulation). Stimulated P cell is shown in cyan in **(A)**. The threshold value of α = 0.05 and consistency criterion of 5 out of 6 were applied for activity detection. **(C)** Cells activated by at least one stimulus type shown in **A,B** were colored according to their putative functional roles similar to Figure 4A. Cyan: sensory neurons; Orange: motor neurons; Green: interneurons; Purple: neurosecretory and partially characterized neurons.

The stimulus-activated neurons were determined based on the same statistical conditions as in the other experiments (α = 0.05 with a consistency value of 5 out of 6). While skin stimulation with medium pressure intensity activated 50 of the 88 analyzed cells, only 22 neurons were found to respond consistently to single P cell stimulation (Table 3, yellow and brown cells in Figures 7A,B). Very similar numbers were obtained for the Friedman's test (*p* < 0.001, Table 3). However, both statistical tests did not identify identical, but substantially overlapping groups of stimulus-activated cells (Table 3, row 4). In comparison between both statistical approaches, the statistics-based method yielded a higher percentage of P cell activated cells being also classified as stimulus-activated for the tactile stimulation (Table 3, column 3), suggesting higher consistency between results. As expected (see section Detection of Statistics-Activated Cells Using Statistics-Based Method), more cells were classified as stimulus-activated when relaxing the consistency criterion to 4 out of 6, but the fraction of approximately twice as many cells being activated by tactile compared to single cell stimulation remained constant (Table 3). Remarkably, the number of cells being classified as stimulus-activated for intracellular P cell stimulation based on the relaxed criterion of 4 consistent responses (37 cells) was still lower than the number of cells found to be activated by tactile stimulation with the more restricted criterion of 5 consistent responses (50 cells).

**Table 3:** Comparison of the number of neurons driven by tactile skin stimulation vs. electrical stimulation of a single P cell.

	**Number of stimulus-activated cells**		
	**P cell**	**Tactile**	**P cell AND Tactile**
Criterion: 5 of 6	22	50	16
Criterion: 4 of 6	37	77	36
Friedman's test	25	49	12
Friedman's test AND Criterion: 5 of 6	12	33	10
Friedman's test AND Criterion: 4 of 6	15	45	12

*Number of stimulus-activated cells in the experiment shown in Figure 7 responding to tactile skin stimulation, single P cell stimulation and to both tactile and P cell stimulation were identified using: statistics-based method with consistency criterion of 5 out of 6 trials (α = 0.05), statistics-based method with consistency criterion of 4 out of 6 trials (α = 0.05), and Friedman's test (p < 0.001) as well as the combination of both tests*.

Using our standard way of identifying stimulus-activated cells (with a consistency value of 5 out of 6), some of these stimulus-activated cells could be assigned to well-known cell types and labeled accordingly in Figure 7C by comparing the VSD image with a standard map of the ganglion (Figure 4A). Additionally, cells were colored to indicate their functional roles in the ganglion suggested by (Wagenaar, 2015) according to Figure 4A. For example, seven putative mechanosensory neurons, identified by their location and relative sizes in the ganglion, were marked in cyan. Similarly, neurosecretory and partially characterized neurons, including Retzius cells, AP cells and cells 251, were colored in purple and motor neurons were marked in orange. The remaining cells, with relatively smaller cell bodies, are putatively interneurons which are activated by either the natural touch stimulation or by single electrical stimulation of the P cell and were marked in green.

16 cells (Table 3, yellow in Figures 7A,B) were active in response to both types of stimulation, mechanical pressure applied to the skin and current injection into a P cell. By comparing the VSD image with a standard map of the ganglion (Figure 4A), some of these cells could be assigned to well-known cell types; e.g., Retzius cells, AP cells, AE cell and cell 251 (cells labeled in Figure 7C). The remaining cells indicated as active in response to both types of stimulation in Figures 7A,B could putatively comprise previously known local bend interneurons (159, 161, 162, 169, 212, 218; Lockery and Kristan, 1990), other neurons receiving synaptic inputs from P cells (204, 208; Kristan et al., 2005) and a premotor neuron (cell 262, Fan et al., 2005).

7 cells (Table 3, and brown in Figure 7A) were exclusively active during P cell current stimulation, including an N cell, which was identified by its location and relatively large, spontaneous spikes. Two cells, cell 251 and one cell in the anterior lateral package, were active during the P cell stimulation condition, but due to their extreme movement, their activity could not be studied in response to tactile stimulation. The remaining cells comprised two unknown neurons in the posterior package, presumably premotor neurons 255 and 261 (Figures 7A,C), as well as cell 62 (a postsynaptic neuron to P cell, Kretzberg et al., 2016) and an interneuron in the anterior lateral package, remained to be identified.

34 cells (Table 3, and brown in Figure 7B) were active during tactile stimulation, but did not show significant activity in response to current stimulation of a single P cell. In addition to 5 mechanoreceptors, the cells specifically responding to tactile skin stimulation putatively comprise one partially characterized cell (251), two motor neurons (one CV and one HE cell), and 27 interneurons, which remained to be identified.

## Discussion

We used voltage-sensitive dye imaging to study the graded activity of local bend interneurons to varied stimulus intensity conditions in two different preparations. In isolated mid-body ganglia a single P cell was stimulated electrically and in semi-intact preparations natural touch stimuli were presented to the skin. The single P cell was stimulated intracellularly with current impulse trains mimicking P cell responses to natural touch stimulations. These experiments allowed us to directly compare the effects of single mechanoreceptor stimulation as the input to the network with those of a population of mechanoreceptors activated by tactile stimulation.

### Evaluation of the methods

All analyses presented in this study relied on the estimation of graded membrane potential changes based on the VSD signals obtained for individual neurons. From intracellular recordings it is known that in many interneurons the slow, graded membrane potential changes are superimposed by small spikes (Lockery and Kristan, 1990; Kretzberg et al., 2016; Pirschel et al., 2018). The amplitudes of these “spikelets” in somatic recordings are usually below 5 mV, making their reliable detection in the optical VSD signals very difficult. Although the big spikes, e.g., of Retzius cells (Figure 1G) and mechanoreceptors were reflected clearly in the VSD signals, not all of the small spikelets could be detected. In particular during touch stimulation, when the VSD signal was prone to additional movement artifacts, the low sampling rate (94.5 Hz) and the low signal-to-noise level of the VSD signals (see Figures 1E,F) only allowed the evaluation of graded membrane potential changes in most of the cells.

Although the SNR and reliability of our VSD imaging traces were comparable to other studies (Frady et al., 2016), not all of the postsynaptic interneurons identified in this study were consistently classified as stimulus-activated cells in all of the preparations. One main reason for this was the limited visibility in VSD images. Out of ~160 neurons on the ventral side of the ganglion, on average only 101 neurons were sufficiently visible for further analyses. Some neurons, especially the ones with smaller cell bodies located below or very close to bigger cells, were not clearly visible in the VSD images with the low spatial resolution of 64 × 128 pixels. Moreover, the removal of the glia sheath, necessary for VSD dye application, caused cell displacement, imposing an additional source of variability between preparations.

The second reason for not detecting all expected cells in all of the experiments was the relatively strict threshold and consistency criteria applied for separating cells' responses from the baseline activity. A cell's activity needed to deviate significantly (α = 0.05) from the baseline activity in at least in 5 out of 6 stimulated trials in exactly the same time frame. The Friedman's test detected a similarly large, overlapping group of stimulus-activated cells (Table 3). Relaxing the criteria of the statistics-based approach led to a larger number of cells classified as stimulus-activated (Figure 2, Table 3), but at the increased risk of false positives.

Thirdly, even though the positions of cell bodies in the ganglion are relatively fixed, they sometimes switch positions. Therefore, more rigorous classification of cell types and mapping of cells across preparations require additional physiological or anatomical evidence.

Moreover, the methodological limitations in removing the two main artifacts of VSD signals, namely bleaching artifact and cells' movements, could have compromised the classification of some stimulus-activated responses. The bleaching decay is a non-linear and stochastic process, which could be approximated with a linear model for our recordings with short duration according to (Fathiazar and Kretzberg, 2015). However, some non-linear bleaching effects might have remained. A similar methodological limitation applied to the optical flow method, which might have failed to eliminate the movement artifact completely in some cases and therefore might have led to misclassification of some cells, even though we excluded the cells with strong movement artifacts from our analyses.

### Interneurons' responses depend weakly on the intensity of intracellular stimulation of a single P cell

We examined the postsynaptic neurons' responses to different spiking patterns of one P cell mimicking typical responses to three different pressure intensities of tactile skin stimulation. P cell stimulation consisted of impulse trains of 5, 7, and 8 current steps, triggering a single spike each, with a timing similar to P cell responses to low, medium and high intesity tactile stimulation. These three different inputs, however, triggered very similar VSD activity maps (Figure 5), suggesting that the response of a single P cell to tactile stimuli of different pressure intensities does not necessarily induce significantly different responses in the majority of the postsynaptic neurons. Despite this similarity, some cells increased their responses consistently with increasing stimulus intensity. For example, the cells with numbers 40–50 showed late responses, occurring several tens of milliseconds after stimulus offset in high intensity condition (Figure 5C). In comparison, these late responses were much weaker during medium intensity condition (Figure 5B). Since a simple feed-forward network would predict interneuron responses to occur much faster than several tens of milliseconds after the last input spike, these findings could indicate that polysynaptic connections or feedback loops lead to delayed interneuron responses to high intensity stimulation.

The postsynaptic neurons identified in this study matched and complemented previous findings. Both Retzius cells and AP cells were relatively big compared to neighboring cells and could be detected easily across preparations. According to the study of Lockery and Kristan (1990), 13 of 17 (8 paired and 1 unpaired) local bend interneurons are located on the ventral side of the ganglion. Although not all of these 13 local bend interneurons were identified in each of our experiments (see section Evaluation of the Methods), all of these cells were found by pooling the stimulus-activated cells of all 6 preparations. Moreover, several cells for which synaptic input from P cells were previously reported, namely, 208, 204, 205 (Kristan et al., 2005), 61, 62 (Kretzberg et al., 2016), and 151 (Marín-Burgin and Szczupak, 2000), were also detected across preparations. Furthermore, our VSD analysis revealed some interneurons in the posterior package as stimulus-activated cells, for which responses to mechanoreceptor inputs were not reported before. These cells could putatively be the pairs of 261, 262, and 255 whose connections to motor neurons were found with dye injections (Fan et al., 2005), additionally linking the sensory layers to motor neurons.

### Tactile stimulation elicits stronger network activity than intracellular stimulation of a single P cell

Comparing the cells activated by a single P cell (Figure 7A) and by tactile skin stimulation (Figure 7B) reveals that many interneurons needed the full sensory input from tactile stimulation to get activated (34 brown cells in Figure 7B). The larger network activity driven by a population of mechanoreceptors compared to a single P cell could be an indication for greater motor responses in the output of the network. These results are consistent with the previous report by Zoccolan and Torre (2002) that a simultaneous stimulation of P and T mechanoreceptors evoked motor responses up to 2-fold greater than a single P mechanoreceptor stimulation. Considering the number of active sensory neurons in response to a local tactile stimulus (4–6: 2 T and 2 P cells with overlapping receptive fields, and also two N cells for stronger stimuli) and in single P cell stimulation (1, 2: the stimulated P cell and maybe a spontaneously active N cell), the larger number of activated interneurons in tactile experiment could be explained with one of the following hypotheses: (1) Some interneurons receive postsynaptic inputs from other sensory neurons than the electrically stimulated P cell, e.g., from T cells. (2) Some interneurons respond to particular activation patterns of a subset of sensory neurons, e.g., as temporal processors or coincidence detectors, responding specifically to concerted inputs from two or more sensory cells. (3) The interneurons were also active in response to single P cell stimulation, but the activity was stronger in the tactile experiment due to the integration of a larger amount of mechanoreceptor inputs, resulting in more depolarized or hyperpolarized values and therefore consistently (in at least 5 out of 6 trials) crossing of threshold values (θ_1_ and θ_2_) than for single P cell stimulation.

These hypotheses need to be evaluated in further VSD and intracellular recording studies stimulating two or more mechanoreceptors in a concerted way in comparison to tactile skin stimulation. These experiments will also help to interpret that some cells, e.g., two unknown neurons in the posterior package (Figure 7A), were active during the P-stimulated condition, but did not show significant activity in response to tactile skin stimulation. This finding could be due to non-linear integration of inputs from different mechanoreceptors, e.g., inhibitory effects of some of the mechanoreceptors.

### Interneurons' responses depend strongly on the intensity of tactile stimulation

Our results indicate that interneurons' responses differed significantly between stimulus conditions, confirming their roles in tactile information processing. More particularly, three distinctive response types were identified in this study. The first group of interneurons (yellow cells in Figure 6H) responded with progressive membrane potential depolarization to increased intensity of tactile stimulation. As the mechanoreceptors also elicit larger numbers of spikes in response to increased stimulus pressure intensities, this group of interneurons could be regarded as slow integrators of presynaptic spikes of sensory neurons, as suggested in our previous study for intracellularly recorded cell 157 (Kretzberg et al., 2016). The same reasoning applies to the smaller group of cells, showing consistently decreasing VSD signals with increasing stimulus intensity (orange cells in Figure 6H). These cells hyperpolarize progressively, suggesting that they integrate inhibitory postsynaptic potentials, induced directly or indirectly by mechanoreceptor spikes.

For the third group of interneurons (magenta cells in Figure 6H), responses differed significantly between stimulus intensity conditions, but without any consistent increase or decrease with increasing stimulus pressure intensities. Hence, these interneurons presumably responded with different temporal response patterns across stimulus conditions. The non-spiking cell 151 is an example of such interneurons, studied previously in detail with intracellular recordings (Marín-Burgin and Szczupak, 2000). This cell receives polysynaptic inputs from mechanosensory P cells and is linked to many motor neurons with rectifying electrical connection. This cell was shown to process sensory signals in the temporal rather than in the amplitude domain (Marín-Burgin and Szczupak, 2000). As reported in our previous study (Kretzberg et al., 2016), cells 159 and 162 are also interneurons with complex temporal response patterns, and were suggested to perform temporal information processing, e.g., as coincidence detectors. Unfortunately, the low signal-to-noise ratio of our VSD signals did not allow a detailed analysis of the temporal structures of the responses. Therefore, further experiments with combined intracellular and VSD recordings are needed to reveal the roles of these types of interneurons in the local bend network of the leech.

Our results suggest that even a single P cell stimulation activates several local bend interneurons, consistent with previous studies (Baljon and Wagenaar, 2015; Frady et al., 2016). However, sensory touch stimuli triggered much more pronounced network activity than a single P cell stimulation, in terms of the number of activated interneurons, the duration of temporal network dynamics, and distinct effects of increasing stimulus intensity on individual cells' responses.

Taken together, our results support the claim (Kretzberg et al., 2016) that the tactile processing in the local bend network is more complex than a simple three-layer feedforward network suggested before (Kristan et al., 2005). The long lasting responses to touch stimuli could be due to involvement of slow synaptic components as were found in mollusks (Snow, 1982; Lieb and Frost, 1997). Late activation of some neurons responding only after approximately 100 ms to stimulus onset indicate the presence of polysynaptic connections in the local bend network (Baljon and Wagenaar, 2015). Temporally concerted inputs from the population of mechanoreceptors representing natural touch stimuli could induce non-linear temporal integration and feedback activity in some of the interneurons' responses (Kretzberg et al., 2016).

In a broader perspective, these experiments on the nervous system of the leech demonstrate that selecting an adequate sensory stimulation is essential to study neuronal dynamics in the context of natural behavior. Based on the finding that spikes from a single P cell can trigger muscle movement (Kristan, 1982; Zoccolan and Torre, 2002), somatic current injection into a single P cell has been assumed to be the adequate trigger for local bend behavior (Baljon and Wagenaar, 2015; Frady et al., 2016; Tomina and Wagenaar, 2017). However, the small number of cells and the relatively easy access to the leech nervous system allowed us to test—and disprove—this assumption experimentally in this study. These results indicate that experimenters should use natural stimulation of sensory organs to make conclusions about behaviorally relevant neuronal information processing–a conclusion that is relevant far beyond the specific case of leech mechanoreception. Nevertheless, the behavioral relevance of the sensory stimulation is arguable also in this study. In nature, a leech would hardly encounter a situation in which a small object suddenly pushes down on the skin, applies constant pressure for 200 ms, and disappears abruptly again. Instead, body movements during unrestrained behavior in natural environments evoke complex activation patterns of all types of mechanoreceptors (Carlton and McVean, 1995). Hence, further studies with more complex, more natural sensory stimulation are needed—and accomplishable for the small nervous system of the leech—to improve our understanding of sensory information processing.

## Author contributions

All authors contributed to planning the study, writing the manuscript and designing the figures. In addition, EF and JK analyzed the data, EF recorded the data and drafted the text and GH established the VSD experiments.

### Conflict of interest statement

The authors declare that the research was conducted in the absence of any commercial or financial relationships that could be construed as a potential conflict of interest.

## References

[B1] Baca S. M. (2005). Location and intensity discrimination in the leech local bend response quantified using optic flow and principal components analysis. J. Neurophysiol. 93, 3560–3572. 10.1152/jn.01263.2004 15689387

[B2] Baca S. M. Marin-Burgin A. Wagenaar D. A. Kristan W. B. (2008). Widespread inhibition proportional to excitation controls the gain of a leech behavioral circuit. Neuron 57, 276–289. 10.1016/j.neuron.2007.11.028 18215624 PMC4084705

[B3] Baljon P. L. Wagenaar D. A. (2015). Responses to conflicting stimuli in a simple stimulus-response pathway. J. Neurosci. 35, 2398–2406. 10.1523/JNEUROSCI.3823-14.2015 25673834 PMC4323524

[B4] Blackshaw S. E. Nicholls J. G. Parnas I. (1982). Expanded receptive fields of cutaneous mechanoreceptor cells after single neurone deletion in leech central nervous system. J. Physiol. 326, 261–268. 10.1113/jphysiol.1982.sp014190 7108791 PMC1251472

[B5] Briggman K. L. (2005). Optical imaging of neuronal populations during decision-making. Science 307, 896–901. 10.1126/science.1103736 15705844

[B6] Briggman K. L. Kristan W. B. (2006). Imaging dedicated and multifunctional neural circuits generating distinct behaviors. J. Neurosci. 26, 10925–10933. 10.1523/JNEUROSCI.3265-06.2006 17050731 PMC6674766

[B7] Burrell B. D. (2017). Leech Mechanosensation. Oxford Research Encyclopedia of Neuroscience. Available online at: http://neuroscience.oxfordre.com/view/10.1093/acrefore/9780190264086.001.0001/acrefore-9780190264086-e-179

[B8] Büschges A. Scholz H. El Manira A. (2011). New moves in motor control. Curr. Biol. 21, R513–R524. 10.1016/j.cub.2011.05.029 21741590

[B9] Carlton T. McVean A. (1995). The role of touch, pressure and nociceptive mechanoreceptors of the leech in unrestrained behaviour. J. Comp. Physiol. A Neuroethol. Sens. Neural Behav. Physiol. 177, 781–791. 10.1007/BF00187637

[B10] Fan R. J. Marin-Burgin A. French K. A. Otto Friesen W. (2005). A dye mixture (Neurobiotin and Alexa 488) reveals extensive dye-coupling among neurons in leeches; physiology confirms the connections. J. Comp. Physiol. A Neuroethol. Sens. Neural Behav. Physiol. 191, 1157–1171. 10.1007/s00359-005-0047-8 16133497

[B11] Fathiazar E. Anemüller J. Kretzberg J. (2016). Statistical identification of stimulus-activated network nodes in multi-neuron voltage-sensitive dye optical recordings, in Engineering in Medicine and Biology Society (EMBC), 2016 IEEE 38th Annual International Conference of the (IEEE), 3899–3903. Available online at: http://ieeexplore.ieee.org/abstract/document/7591580/ (Accessed May 30, 2017). 10.1109/EMBC.2016.7591580 28269138

[B12] Fathiazar E. Kretzberg J. (2015). Estimation of neuronal activity based on voltage-sensitive dye imaging in a moving preparation, in Engineering in Medicine and Biology Society (EMBC), 2015 37th Annual International Conference of the IEEE (IEEE), 6285–6288. Available online at: http://ieeexplore.ieee.org/abstract/document/7319829/ (Accessed May 30, 2017). 10.1109/EMBC.2015.7319829 26737729

[B13] Frady E. P. Kapoor A. Horvitz E. Kristan W. B. Jr. (2016). Scalable semisupervised functional neurocartography reveals canonical neurons in behavioral networks. Neural Comput. 28, 1453–1497. 10.1162/NECO_a_00852 27348420

[B14] Hollander M. Wolfe D. A. Chicken E. (2013). Nonparametric Statistical Methods, 3rd Edn. Hoboken, NJ: John Wiley & Sons.

[B15] Kretzberg J. Pirschel F. Fathiazar E. Hilgen G. (2016). Encoding of tactile stimuli by mechanoreceptors and interneurons of the medicinal leech. Front. Physiol. 7:506. 10.3389/fphys.2016.00506 27840612 PMC5083904

[B16] Kristan W. B. (1982). Sensory and motor neurones responsible for the local bending response in leeches. J. Exp. Biol. 96, 161–180.

[B17] Kristan W. B. Calabrese R. L. Friesen W. O. (2005). Neuronal control of leech behavior. Prog. Neurobiol. 76, 279–327. 10.1016/j.pneurobio.2005.09.004 16260077

[B18] Lewis J. E. Kristan W. B. (1998). A neuronal network for computing population vectors in the leech. Nature 391, 76–79. 10.1038/34172 9422507

[B19] Lieb J. R. Frost W. N. (1997). Realistic simulation of the aplysia siphon-withdrawal reflex circuit: roles of circuit elements in producing motor output. J. Neurophysiol. 77, 1249–1268. 10.1152/jn.1997.77.3.1249 9084594

[B20] Lockery S. R. Kristan W. B. (1990). Distributed processing of sensory information in the leech. II. Identification of interneurons contributing to the local bending reflex. J. Neurosci. 10, 1816–1829. 2355252 10.1523/JNEUROSCI.10-06-01816.1990 PMC6570306

[B21] Marin-Burgin A. Kristan W. B. French K. A. (2008). From synapses to behavior: development of a sensory-motor circuit in the leech. Dev. Neurobiol. 68, 779–787. 10.1002/dneu.20551 18383550

[B22] Marín-Burgin A. Szczupak L. (2000). Processing of sensory signals by a non-spiking neuron in the leech. J. Comp. Physiol. A 186, 989–997. 10.1007/s003590000152 11138800

[B23] Miller E. W. Lin J. Y. Frady E. P. Steinbach P. A. Kristan W. B. J. Tsien R. Y. (2012). Optically monitoring voltage in neurons by photo-induced electron transfer through molecular wires. Proc. Natl. Acad. Sci. U.S.A. 109, 2114–2119. 10.1073/pnas.1120694109 22308458 PMC3277584

[B24] Milliken G. A. Johnson D. E. (2009). Analysis of Messy Data, Vol. 1, Designed Experiments, 2nd Edn. New York, NY: CRC Press.

[B25] Muller K. J. Scott S. A. (1981). Transmission at a “direct” electrical connexion mediated by an interneurone in the leech. J. Physiol. 311, 565–583. 6267257 10.1113/jphysiol.1981.sp013605 PMC1275430

[B26] Nicholls J. G. Baylor D. A. (1968). Specific modalities and receptive fields of sensory neurons in CNS of the leech. J. Neurophysiol. 31, 740–756. 10.1152/jn.1968.31.5.740 5711143

[B27] Nicholls J. G. Purves D. (1970). Monosynaptic chemical and electrical connexions between sensory and motor cells in the central nervous system of the leech. J. Physiol. 209, 647–667. 10.1113/jphysiol.1970.sp009184 5499801 PMC1395546

[B28] Pearson K. G. (1993). Common principles of motor control in vertebrates and invertebrates. Annu. Rev. Neurosci. 16, 265–297. 10.1146/annurev.ne.16.030193.001405 8460894

[B29] Pirschel F. Hilgen G. Kretzberg J. (2018). Effects of touch location and intensity on interneurons of the leech local bend network. Sci. Rep. 8:3046. 10.1038/s41598-018-21272-6 29445203 PMC5813025

[B30] Pirschel F. Kretzberg J. (2016). Multiplexed population coding of stimulus properties by leech mechanosensory cells. J. Neurosci. 36, 3636–3647. 10.1523/JNEUROSCI.1753-15.2016 27030751 PMC6601736

[B31] Snow R. W. (1982). Characterization of the synaptic actions of an interneuron in the central nervous system of Tritonia. J. Neurobiol. 13, 251–266. 10.1002/neu.480130306 7077321

[B32] Taylor A. L. Cottrell G. W. Kleinfeld D. Kristan W. B. (2003). Imaging reveals synaptic targets of a swim-terminating neuron in the leech CNS. J. Neurosci. 23, 11402–11410. Available online at: http://www.jneurosci.org/content/23/36/11402.long 14673004 10.1523/JNEUROSCI.23-36-11402.2003 PMC6740517

[B33] Thomson E. E. Kristan W. B. (2006). Encoding and decoding touch location in the leech CNS. J. Neurosci. 26, 8009–8016. 10.1523/JNEUROSCI.5472-05.2006 16870746 PMC6674225

[B34] Tomina Y. Wagenaar D. A. (2017). A double-sided microscope to realize whole-ganglion imaging of membrane potential in the medicinal leech. Elife 6:e29839. 10.7554/eLife.29839 28944754 PMC5656430

[B35] Wagenaar D. A. (2015). A classic model animal in the 21st century: recent lessons from the leech nervous system. J. Exp. Biol. 218, 3353–3359. 10.1242/jeb.113860 26538172

[B36] Zoccolan D. Torre V. (2002). Using optical flow to characterize sensory-motor interactions in a segment of the medicinal leech. J. Neurosci. 22, 2283–2298. Available online at: http://www.jneurosci.org/content/22/6/2283.long 11896168 10.1523/JNEUROSCI.22-06-02283.2002 PMC6758248

